# Immunogenic cell death-related classifications in breast cancer identify precise immunotherapy biomarkers and enable prognostic stratification

**DOI:** 10.3389/fgene.2022.1052720

**Published:** 2022-11-10

**Authors:** Xue Wang, Hailiang Huang, Xijian Liu, Jiuwei Li, Lu Wang, Ling Li, Yaxing Li, Tao Han

**Affiliations:** ^1^ Pharmacology of Traditional Chinese Medical Formulae, College of Traditional Chinese Medicine, Shandong University of Traditional Chinese Medicine, Jinan, China; ^2^ College of Rehabilitation Medicine, Shandong University of Traditional Chinese Medicine, Jinan, China; ^3^ College of Medical, Shandong University of Traditional Chinese Medicine, Jinan, China; ^4^ Office of Academic Research, Shandong University of Traditional Chinese Medicine, Jinan, China

**Keywords:** immunogenic cell death, breast cancer, tumor microenvironment, immunotherapy, risk prognosis

## Abstract

**Background:** Immunogenic cell death (ICD) remodels the tumor immune microenvironment, plays an inherent role in tumor cell apoptosis, and promotes durable protective antitumor immunity. Currently, appropriate biomarker-based ICD immunotherapy for breast cancer (BC) is under active exploration.

**Methods:** To determine the potential link between ICD genes and the clinical risk of BC, TCGA-BC was used as the training set and GSE58812 was used as the validation set. Gene expression, consistent clustering, enrichment analysis, and mutation omics analyses were performed to analyze the potential biological pathways of ICD genes involved in BC. Furthermore, a risk and prognosis model of ICD was constructed to evaluate the correlation between risk grade and immune infiltration, clinical stage, and survival prognosis.

**Results:** We identified two ICD-related subtypes by consistent clustering and found that the C2 subtype was associated with good survival prognosis, abundant immune cell infiltration, and high activity of immune biological processes. Based on this, we constructed and validated an ICD risk and prognosis model of BC, including ATG5, HSP90AA1, PIK3CA, EIF2AK3, MYD88, IL1R1, and CD8A. This model can effectively predict the survival rate of patients with BC and is negatively correlated with the immune microenvironment and clinical stage.

**Conclusion:** This study provides new insights into the role of ICD in BC. The novel classification risk model based on ICD in BC established in this study can aid in estimating the potential prognosis of patients with BC and the clinical outcomes of immunotherapy and postulates targets that are more useful in comprehensive treatment strategies.

## Background

Immunogenic cell death (ICD) is a special type of regulatory cell death that can remodel the adaptive immunity in the tumor microenvironment and establish immune memory, enabling patients to obtain long-term clinical benefits (Galluzzi et al., 2020). ICD differs from other cell apoptosis in that it is defined by the release of damage-associated molecular patterns. In the case of tumor cells, or during the course of infection, dead cells can stimulate a robust adaptive immune response against the changed autoantigens/cancer derived new epitopes or pathogen derived antigens. This increases the exposure of endogenous adjuvants or intracellular molecules and triggers adaptive immunity through antigen presentation, increasing the recruitment of T cells and promoting the entire immune cycle mechanism (Hernández Á. et al., 2021). The unique anticancer immune regulation ability of ICD provides new hope to the current limitations of cancer therapy, especially for breast cancer (BC).

Breast cancer is characterized by low immunogenicity due to low mutation rates and reduced lymphocyte infiltration; therefore, the immunotherapy response in BC remains modest and unpredictable (Hanna and Balko, 2021). Research has found the number of infiltrating lymphocytes, especially CD4 and CD8 T cells and dendritic cells, in the tumor immune microenvironment can be used as a prognostic indicator for patients with BC (Li et al., 2021). Therefore, increasing immune cell infiltration in the tumor microenvironment is predicted to be an effective way to improve the outcomes of BC immunotherapy.

Immunogenic cell death can release relevant signals through adenosine triphosphate during the development of BC and recruit immune cells to infiltrate the tumor site (Reyes-Ruiz et al., 2021). At present, it has been confirmed that ICD in BC can be promoted by multiple pathways and targets. Anthracyclines, as first-line chemotherapy drugs for BC, can promote ICD, specifically by activating the NLRP3 inflammasome to induce adaptive immunity (Ghiringhelli et al., 2009). In addition, they can induce high mobility group protein B1 (HMGB1) to be passively released from dead cells in the ICD damage-associated molecular patterns. Clinical studies have shown that in patients with BC treated with anthracyclines, the level of HMGB1 increases, which predicts better survival prognosis (Stoetzer et al., 2013). This also suggests that anti-cancer treatment not only eliminates cancer cells in the traditional way but also induces ICD targeting related biomarkers to trigger anti-tumor immunity in patients with BC and promotes the extension of cancer immune cycle memory. Moreover, heat shock proteins (HSPs) have been proven to be associated with the ICD process and have the main function of repairing protective proteins. Therefore, their presence in cell components is recognized as a beneficial anti-apoptotic component (Wu et al., 2017). HSPs can recruit dendritic cells through different transmitters and present antigens to T cells, thus, increasing tumor immunogenicity (Albakova et al., 2021). These exciting research findings have aroused an upsurge of ICD-related research among scholars, but there is limited knowledge on effective and accurate ICD biomarkers in BC, which prompted us to investigate them in depth.

In this study, we aimed to identify biomarkers related to ICD in BC and to explore their potential pathogenesis in patients with BC. Construction of an ICD risk model is needed to evaluate the prognosis, immune microenvironment, and clinical treatment of patients with BC. Therefore, this study aimed to provide a theoretical basis for the immunotherapy of patients with BC.

## Materials and methods

### Datasets

For the training set, the transcriptome information of a total of 1211 BC cases was downloaded from The Cancer Genome Atlas database (TCGA, https://portal.gdc.cancer.gov/), including 1098 BC samples and 113 normal samples (Ganini et al., 2021). For the validation set, the GSE58812 microarray gene chip from the Gene Expression Omnibus database (GEO, www.ncbi.nlm.nih.gov/geo/, GEO accession: GSE58812, Platforms: GPL570) was used, which comprised 107 patients with complete clinical information (Albaradei et al., 2021). Gene mutations and matching clinicopathological data for TCGA-BC dataset were also obtained from TCGA database. All the data involved in this study were obtained from an open platform, so no ethical permission was required.

### Identification of the immunogenic cell death subtypes

ConsensusClusterPlus was used for combining BC mRNA expression and ICD-related genes for cluster analysis. ConsensusClusterPlus can visualize the number of unsupervised clusters in sample data and is widely used in cancer research (Wilkerson and Hayes, 2010). Eighty percent of the samples were resampled for 10 repetitions according to the area under the Consensus Cumulative Distribution Function curve, K-value, and intra-group consistency to ensure the stability of the results.

### Analysis of differentially expressed genes

The differentially expressed genes (DEGs) between clustering subtypes were analyzed by the t. test function in R software to explore the potential differences between the two ICD subtypes. The screening criteria for DEGs was determined as the adjusted *p* < 0.05 and |fold change| > 1.5. Next, we further enriched the DEGs to compare the differential signaling pathways and biological effects between the different ICD groups. Gene ontology (GO) and Kyoto Encyclopedia of Genes and Genomes (KEGG) enrichment analyses were based on the premise of *p* < 0.05.

### Gene set enrichment analysis

The samples were divided into two subtypes based on the ICD gene expression. Gene set enrichment analysis (GSEA) was used to evaluate differences in related pathways and molecular mechanisms between the two subtypes. GSEA is an effective method that is commonly used to analyze the underlying biological processes of DEGs in samples (Hung et al., 2012). The minimum gene set was set to 5, the maximum gene set to 5,000, and re-sampling to 1,000 times. |NES| > 1, *p* < 0.05, and FDR <0.25 were considered statistically significant.

### Somatic mutation analysis

Somatic mutation analysis is crucial for identifying driver genes in cancer, which helps to reveal the potential internal causes of cancer occurrence and promotes the clinical progress of targeted therapy (Dressler et al., 2022). Somatic mutation data of 1,071 patients with BC were obtained from TCGA to explore whether genetic structural changes occurred between the different clusters. The chi-squared test was used to evaluate the difference in gene mutation frequency in each group of samples, and the mutated genes were visualized using waterfall plots.

### Immune infiltration between two immunogenic cell death subtypes

Immuno-Oncology Biological Research (IOBR) analysis can use multi-omics data to analyze the relationship between tumors and immunity (Zeng et al., 2021). Based on our expression profile, we used the CIBERSORT (Newman et al., 2015), ESTIMATE (Yoshihara et al., 2013), and TIMER (Li et al., 2016) methods to calculate the three immune infiltrating cell scores of each sample in the two cluster subtypes.

### Construction of the immunogenic cell death risk model

The Cox proportional hazard model with the least absolute shrinkage and selection operator for variable selection (Lasso-Cox) is an accurate and effective feature selection and risk prediction algorithm (Wang and Liu, 2020). We used the glmnet package to integrate survival time, survival status, and gene expression data, and used Lasso-Cox for regression analysis. In addition, we set up a 10-fold cross validation test to obtain the optimal model. The correlation between the risk model score, tumor immune infiltration microenvironment, and clinical stage of patients with BC was further analyzed.

## Results

### Consensus clustering identified two immunogenic cell death-associated subtypes

**FIGURE 1:**
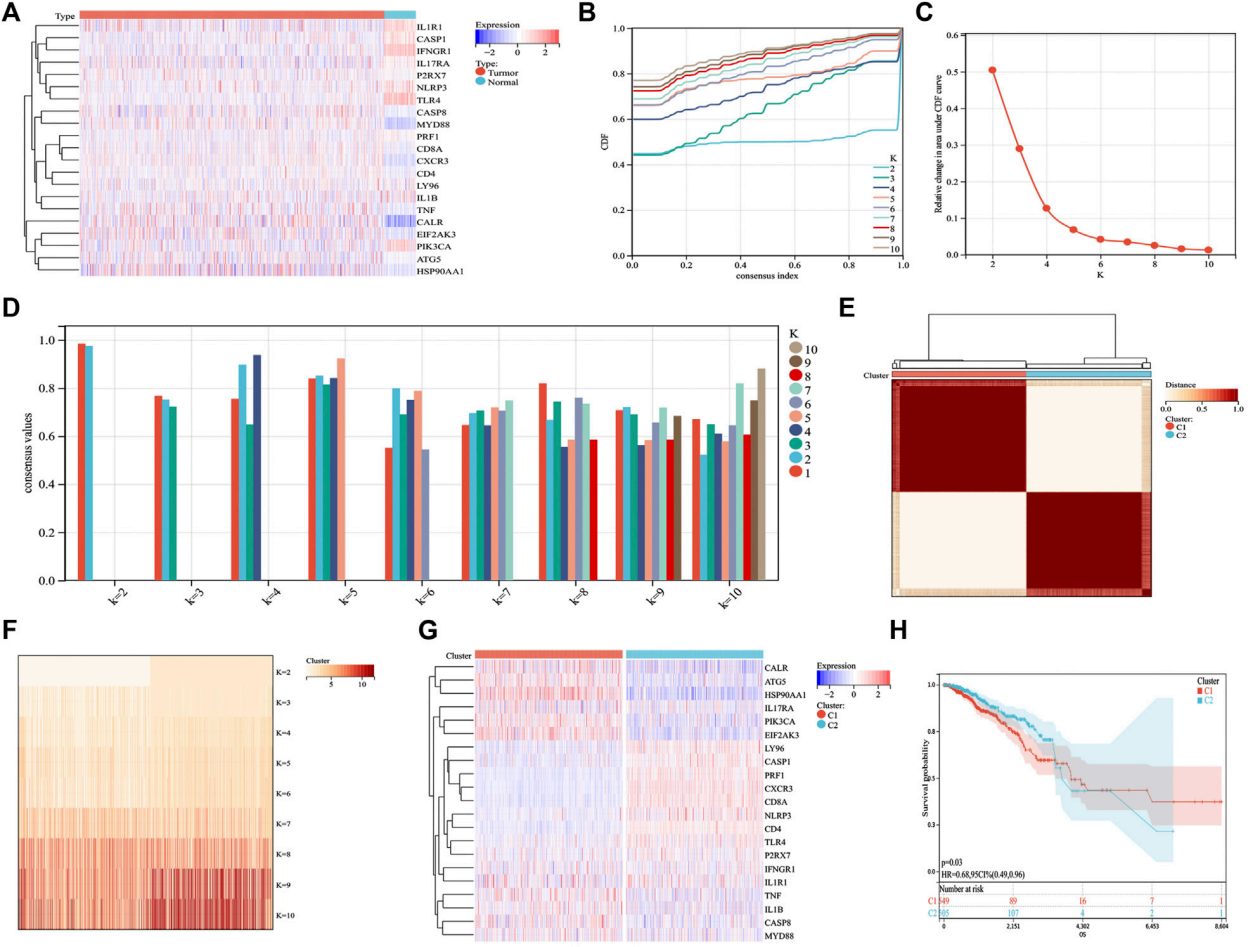
Identification of ICD subtypes by consistent clustering. **(A)** 21 ICD genes were expressed in BC samples, including tumor and normal samples. **(B,C)** Cluster analysis cumulative distribution function (CDF), indicating the area under the curve and delta decreasing trend when *k* = 2–10. **(D)** Sample cluster consistency diagram, showing that when *k* = 2, consensus values are best. **(E,F)** Heatmap depicting the best scheme for consensus clustering (*k* = 2) for 21 genes in 1,098 BC samples. **(G)** Heatmap of the expression of 21 ICD genes in two subtypes. Red represents high expression; blue represents low expression. **(H)** Kaplan–Meier curves of the overall survival in two subtypes; the C2 group had better prognostic outcomes than the C1 group. *p* = 0.03.

Based on published ICD-related literature (Garg et al., 2016; Galluzzi et al., 2020), we identified 21 ICD-related genes (*ATG5*, *CALR*, *CASP1*, *CASP8*, *CD4, CD8A*, *CXCR3*, *EIF2AK3*, *HSP90AA1*, *IFNGR1*, *IL17RA*, *IL1B*, *IL1R1*, *LY96*, *MYD88*, *NLRP3*, *P2RX7*, *PIK3CA*, *PRF1*, *TLR4*, *TNF*) and analyzed their expression differences between BC and normal samples. ICD genes, such as *CALR*, *CASP8*, *P2RX7*, *MYD88*, *CD8A*, *CXCR3*, *CD4*, *TNF*, *ATG5*, and *HSP90AA1*, were found to be abnormally highly expressed in BC samples (Figure 1A). Further cluster analysis revealed that when K = 2, the highest number of clusters appeared in the average consistency within the group. Therefore, the two subtypes in BC samples showed different ICD gene expression (Figures 1B–G). We further explored whether different subtypes of C1 and C2 affected the survival of patients with BC. The results are shown in Figure 1H, where C2 indicates better clinical results.

### Differential gene expression and enrichment analysis of immunogenic cell death subtypes

**FIGURE 2:**
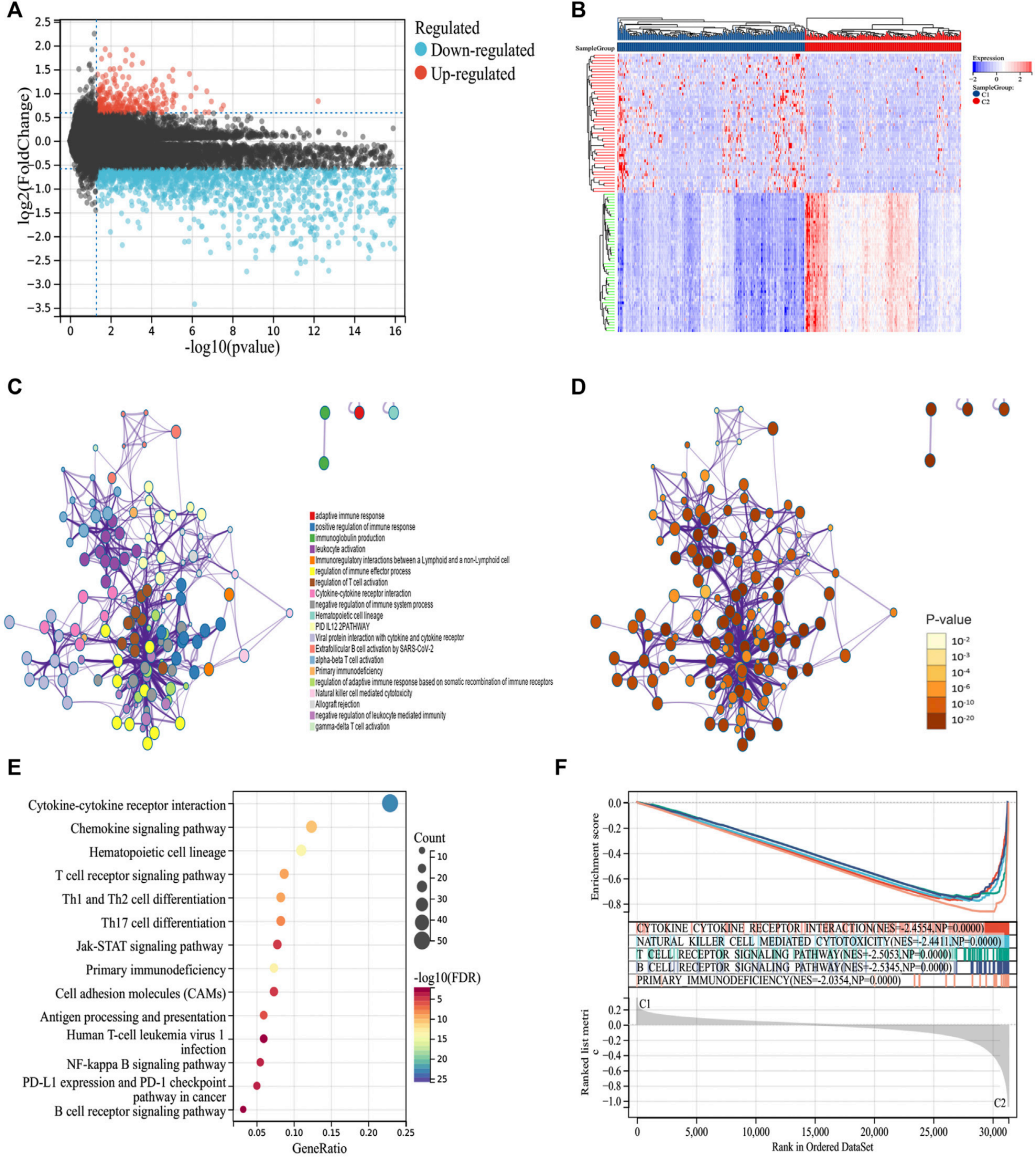
Identifying differentially expressed genes (DEGs) and biological pathways between the two subtypes. **(A)** Volcano map showing the distribution of DEGs between C1 and C2, where red represents high expression and blue represents low expression. **(B)** Heatmap showing the expression of DEGs between the two subtypes. **(C,D)** Metascape enrichment network displaying the gene function enrichment of DEGs, where each cluster annotation is color coded. **(E)** A bubble plot showing the enrichment of signal pathways of DEGs. **(F)** GSEA analysis identifying the enrichment of different pathways between C1 and C2.

To explore the molecular mechanism of the difference in prognosis between the two ICD subtypes, we first identified 276 upregulated and 2096 downregulated DEGs (Figures 2A,B). Further enrichment analysis showed that DEGs were significantly involved in immune-related processes, such as positive regulation of the immune response, immunoglobulin production, leukocyte activation, immunoregulatory interactions between lymphoid and non-lymphoid cells, regulation of immune effector processes, and regulation of T cell activation (Figures 2C–E). Furthermore, we compared the signal pathways between the two subtypes using GSEA and found that the DEGs were closely related to the immune pathway and were significantly enriched in group C2, indicating that group C2 is related to immunity (Figure 2F). Thus, the molecular mechanism revealed the potential reason for the better prognosis of group C2.

### Comparison of somatic mutations and the tumor microenvironment among immunogenic cell death subtypes

**FIGURE 3:**
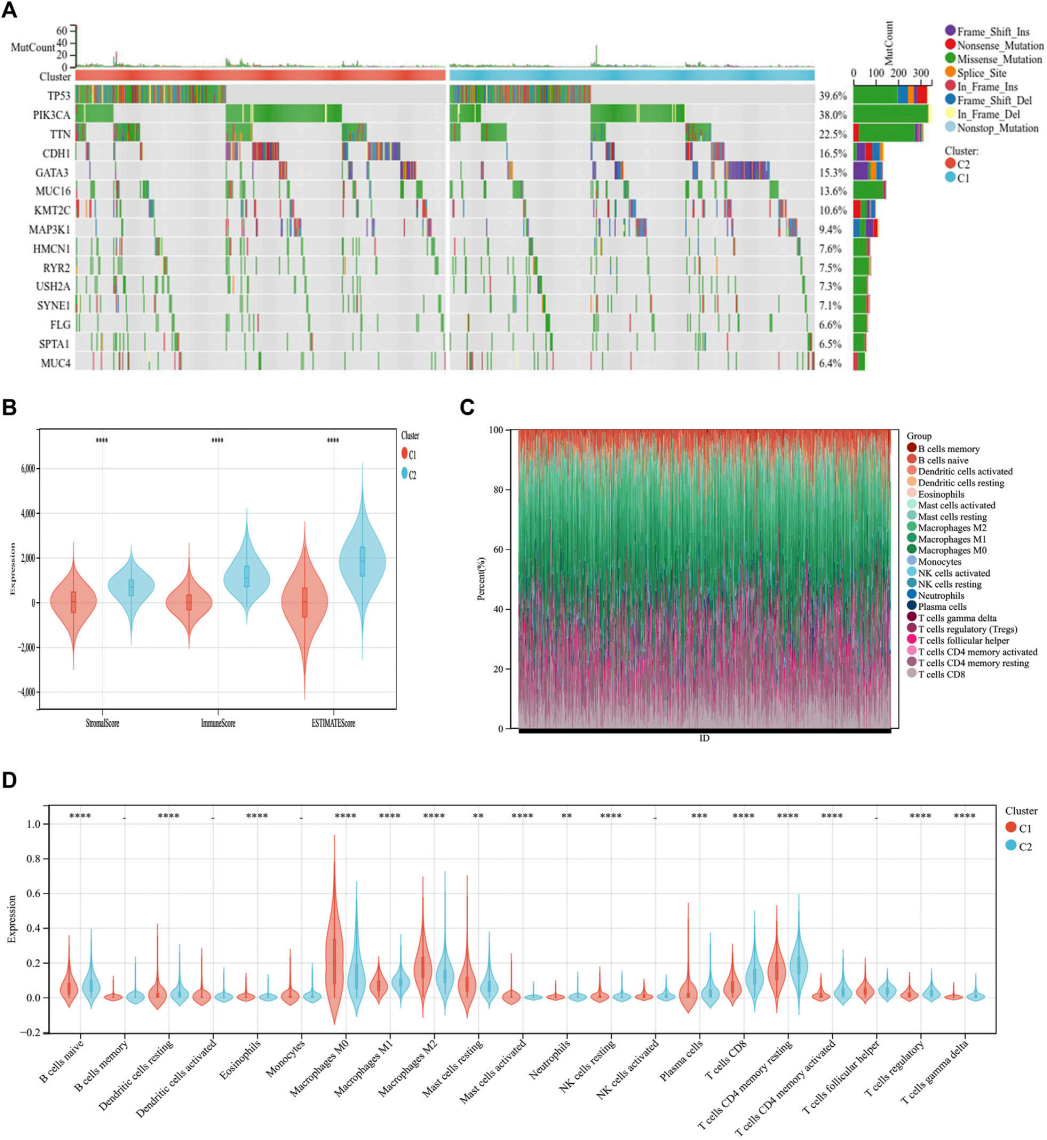
Differential somatic mutations and immune infiltration in two ICD subtypes. **(A)** Gene mutation map showing that the 10 most commonly mutated genes in BC differ in mutations between the C1 and C2 groups. **(B)** ESTIMATE violin plot showing the differences in the stromal and immune cells infiltrating C1 and C2. **(C)** The relative proportions of immune-infiltrating types in C1 and C2. **(D)** CIBERSORT violin plot showing multiple differences in immune cell enrichment between the two ICD subtypes. *p < 0.05, **p < 0.01, ***p < 0.001, ****p < 0.0001.

Through somatic mutation analysis, we noted a mutation difference between C1 and C2 (Figure 3A). In the common tumor-mutated genes *TP53*, *PIK3CA*, *TTN*, *CDH1*, and *GATA3*, the mutation frequencies among the two subtypes were different. The frequency of mutations in *TP53*, *PIK3CA*, *TTN*, and *CDH1* was higher in group C2 than in group C1. The latest research reports that ICD can promote an antitumor immune microenvironment (Iulianna et al., 2022; Mishchenko et al., 2022). Therefore, we explored the tumor microenvironment of the two subtypes. First, the immune score, matrix score, and estimated score of group C2 was higher than those of group C1 (Figure 3B). Second, we continued to explore the differences in immune cell infiltration between the two subtypes using CIBERSORT. The infiltration expression of immune cells in C2 was higher than that in C1, including naive B cells, macrophages, plasma cells, CD8 T cells, and CD4 T cells (Figures 3C,D). This indicates that the C2 group has a better prognosis and, simultaneously, the immune cell infiltration is higher than that in C1.

### Construction of the immunogenic cell death risk model

**FIGURE 4:**
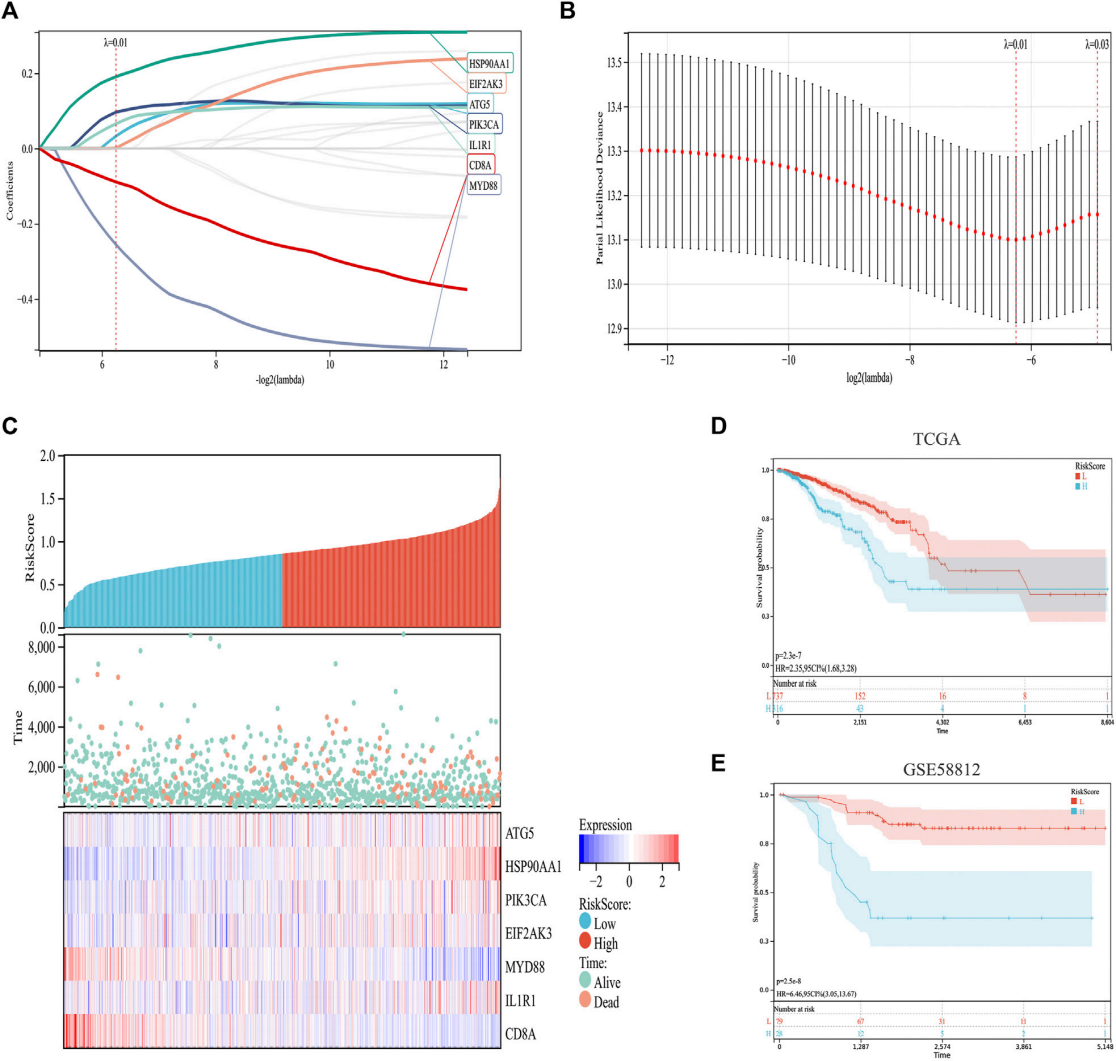
Construction and validation of an ICD risk model. **(A,B)** Lasso-Cox analysis identifies seven ICD genes associated with overall survival in BC. **(C)** Prognostic heatmap showing the relationship between different risk scores of the ICD risk model, patient survival events, and changes in gene expression. **(D,E)** Survival analysis plot showing the risk model has valid prognostic significance in both the TCGA (*p* = 2.3 × 10^−7^) and GSE58812 (*p* = 2.5 × 10^−8^) cohorts.

Based on previous analysis, we established a prediction model based on ICD-related genes. Specifically, by integrating the survival time, survival status, and gene expression data, a regression analysis was carried out using Lasso-Cox (Figures 4A,B). We set the lambda value to 0.0131770633945094 and obtained seven biomarkers (Figures 4A–C). The model formula is as follows:

$$\begin{array}{l} Risk\;Score=0.0326363881498623*ATG5+0.190693483244363*HSP90AA1+\\ 0.0965024720502192*PIK3CA+0.000619152780442697*EIF2AK3-0.25550716880708*MYD88+\\ 0.0655640833683533*IL1R1-0.0890578092004148*CD8A \end{array}$$

In addition, the survival status of patients with BC was analyzed according to the risk score, and the survival status in the low-risk cohort was found to be much higher than that in the high-risk cohort (Figure 4C). Further survival analyses verified this hypothesis. In TCGA set, the low-risk cohort predicted a better survival prognosis (Figure 4D), and the survival results of the validation set showed the same trend (Figure 4E).

### Relationship between the immunogenic cell death risk model, tumor microenvironment, and clinical indicators

**FIGURE 5:**
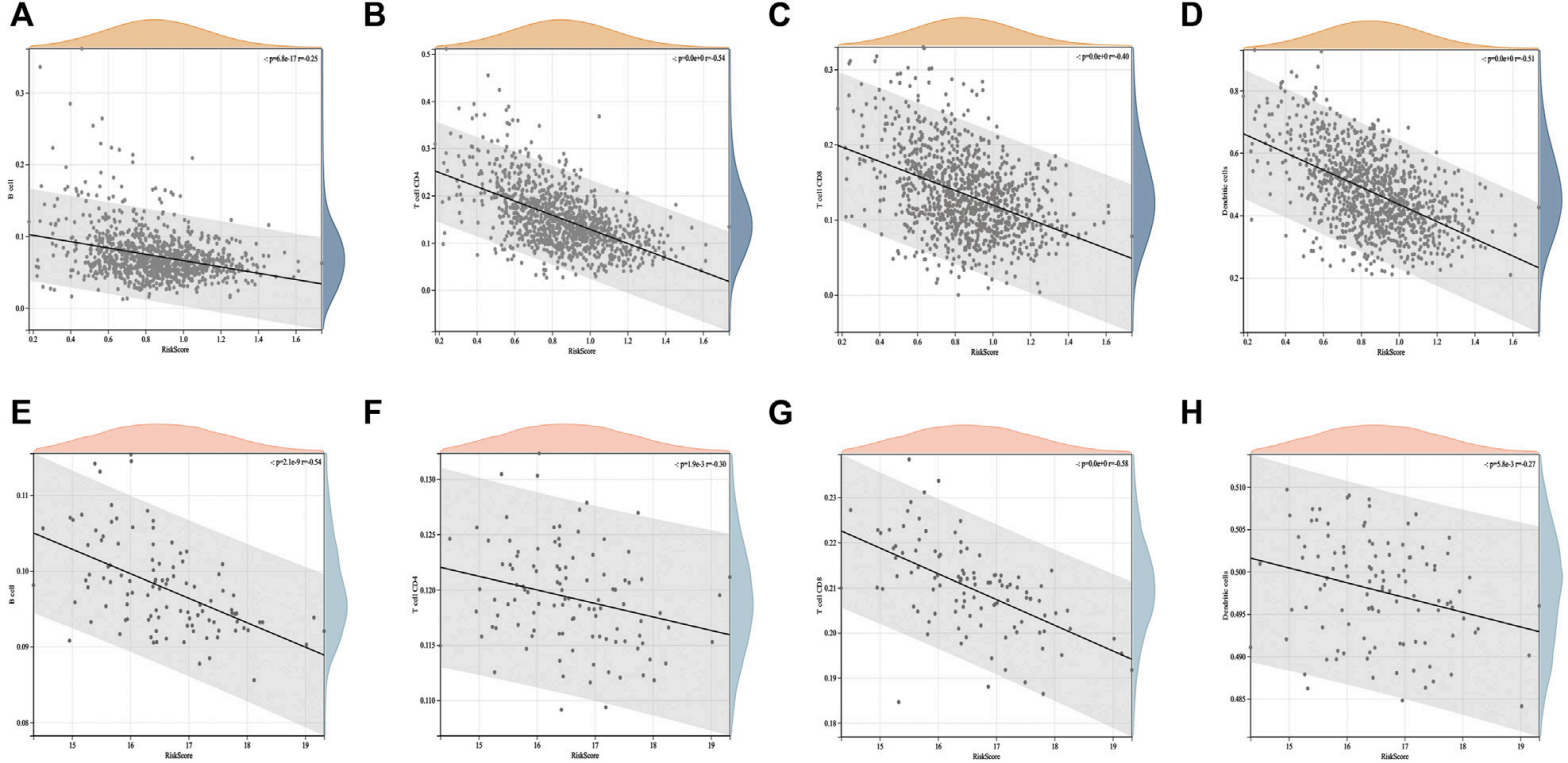
Correlation between the ICD risk model and tumor microenvironment. The scatter plots indicate that the ICD risk score is significantly negatively correlated with B cells, CD4 T cells, and CD8 T cells **(A–D)**, and further validation of the GEO cohort showed the same trend **(E–H)**.

We conducted an in-depth analysis of the correlation between the ICD risk score and tumor microenvironment. The results showed that the ICD risk score was significantly negatively correlated with the number of B cells, CD4 T cells, CD8 T cells, and dendritic cells in BC (Figures 5A–D). This suggests that the number of immune cell infiltrations decreased with an increase in the ICD risk score. The higher the ICD risk score, the worse the immune infiltration status in the BC tumor microenvironment. This was further confirmed in the GSE58812 validation set (Figures 5E–H).

**FIGURE 6:**
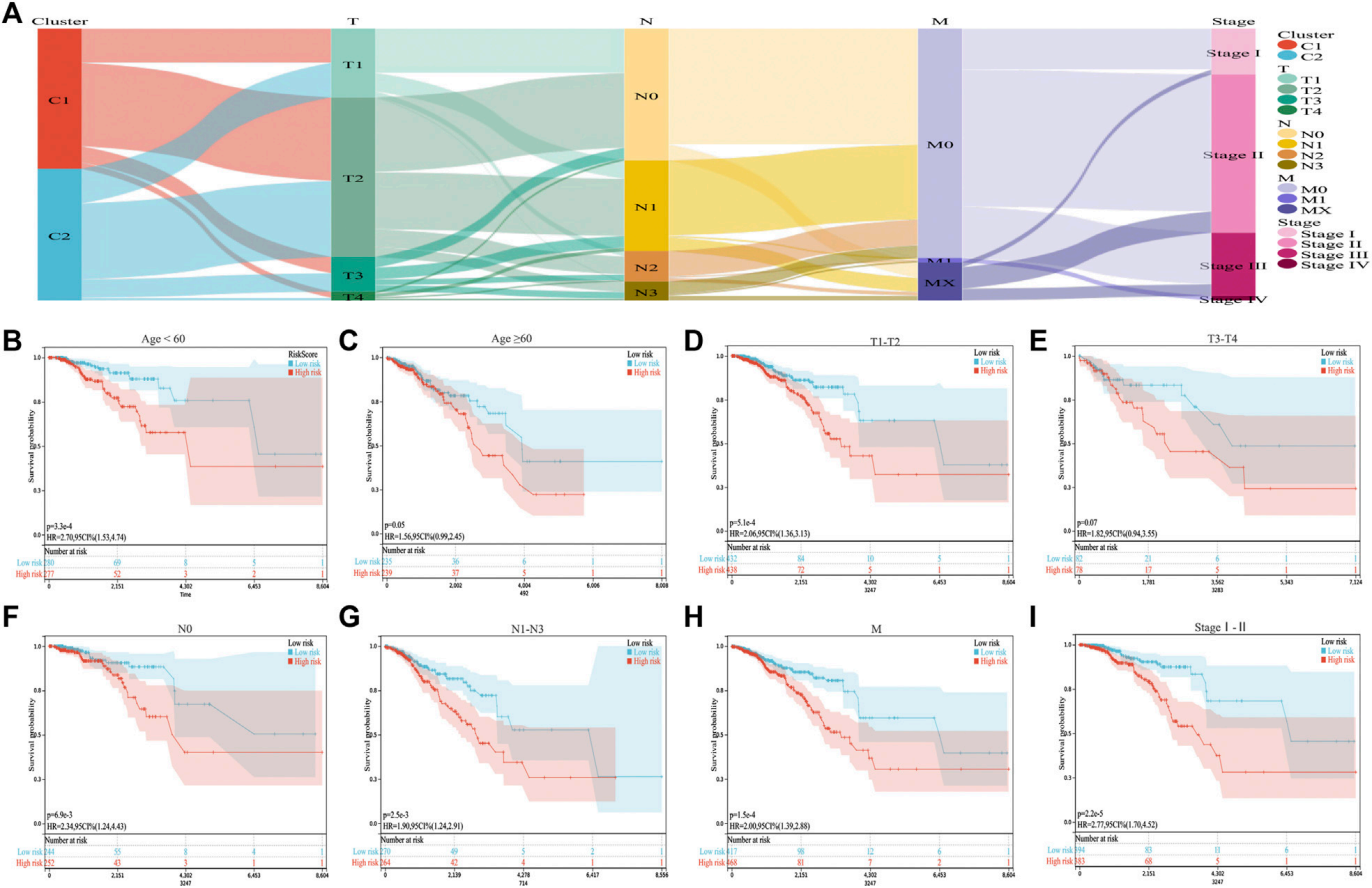
Correlation between the ICD risk model and clinical prognosis. **(A)** Sankey diagram showing the distribution of BC clinical information in the two subtypes. **(B–I)** Survival prognosis diagram, which analyzes the relationship with the ICD risk score with regards to age, tumor node metastasis classification, and clinical stage, which all show that a low ICD risk score indicates a better clinical prognosis.

We combined the ICD risk score with the clinical characteristics of patients with BC and analyzed potential prognostic indicators. The available clinical information included age, tumor node metastasis classification, and clinical stage, as shown in Figure 6A. We evaluated the relationship between patient age, tumor size, lymph node metastasis, distant metastasis, and clinical stage using the ICD risk score (Figures 6B–I). The results showed a consistent trend that patients with low ICD risk scores were predicted to have better clinical outcomes.

## Discussion

As a form of cell death that can cause an immune response, ICD can stimulate or release immunoregulatory properties while tumor cells die and multiple immune pathways to promote immune responses (Sansone et al., 2021). The current low response rate and multiple side effects of tumor immunotherapy can hopefully be solved by inducing ICD (Li et al., 2022). ICD can not only reverse the microenvironment of tumor immunosuppression but also improve the sensitivity of immunotherapy. Therefore, according to the urgent need for tumor immunotherapy, the identification of ICD-related biomarkers in patients with BC can serve as an effective clinical treatment. Based on this, this study explored the potential relationship between ICD-related genes and BC and found that ICD-related genes were closely related to the survival prognosis, tumor microenvironment, and clinical characteristics of patients with BC. Specifically, we constructed and validated a prognostic risk model for seven ICD-related genes and used it to classify patients with BC into high- and low-risk cohorts. It is worth mentioning that this risk model score showed a high predictive value for BC survival prognosis and showed a negative correlation with the tumor microenvironment and clinical characteristics.

In this study, BC samples were divided into subgroups C1 and C2 based on the consistent clustering analysis of ICD-related genes. The results showed that the two subgroups could effectively distinguish between the survival and prognosis of patients with BC. GO and KEGG analyses showed that the DEGs in the two subgroups were significantly enriched in the adaptive immune response pathways, processes regulating the immune effect, and immune-related cytokine signaling. DEGs can interfere with the progression of tumors *via* two mechanisms: innate and adaptive immunity (Sun et al., 2020). GSEA further elucidated the presence of differential regulation in the C1 and C2 subgroups. The results showed that immune-related signaling pathways, for example, natural killer cell-mediated cytotoxicity and the T cell receptor signaling pathway, were significantly enriched in the C2 group. This could also potentially explain the reason for better survival and prognosis of the C2 group of patients with BC at the biological level. The differential expression of ICD genes in patients with BC prompted us to construct a prognostic risk model of ICD-related genes.

Initially, 21 confirmed ICD genes were studied, and 7 were found to be significantly associated with the survival and prognosis of patients with BC. Among them, the oncogenic genes were *PIK3CA* (Hou et al., 2022; Ouedraogo et al., 2022), *EIF2AK3* (Chen et al., 2019), and *MYD88* (Chen et al., 2015), and the immune-related genes were *ATG5* (Park et al., 2022), *HSP90AA1* (Lin et al., 2020; Liu et al., 2021), *IL1R1* (Dagenais et al., 2017; Tulotta et al., 2021), and *CD8A* (Hu et al., 2021), all of which have been found to be related to the occurrence and development of BC. Cho et al. (2022) found using immunohistochemistry that *PIK3CA* mutations in patients with BC receiving adjuvant chemotherapy could cause poor survival and prognosis. Chen et al. analyzed the relationship between the expression of *MYD88* and clinical features in the pathological tissues of 60 patients with BC and found that the expression of *MYD88* in tumor tissues was significantly higher than that in adjacent normal tissues, and protein expression was significantly correlated with adverse clinical features (Park et al., 2022). In addition, the latest study on *HSP90AA1* shows that it not only is a pro-oncogene in BC but also plays a regulatory role in the immune microenvironment (Lin et al., 2020). *HSP90AA1* regulates the infiltration of immune cells in BC and has a potential relationship with T cells, neutrophils, macrophages, and dendritic cells. This study also identified *HSP90AA1* as a new target for BC immunotherapy.

Immunogenic cell death induced by anticancer drugs may trigger individual BC patients to acquire anti-tumor immune memory and promote the treatment process of BC. This also inspired many ICD biomarkers and therapeutic drugs in BC in-depth exploration. As early as 2013, researchers conducted clinical trials on 51 BC patients, and found that *HMGB1* and *RAGE* are key biomarkers to promote the process of ICD in patients (Stoetzer et al., 2013). High expression of *HMGB1* and low expression of *RAGE* can effectively predict better therapeutic response. In addition, Li et al. (2020) conducted an experimental study on the ICD of BC *in vitro*. The results showed that inhibiting the expression of the ICD-related genes *CDK12/13* could release *HMGB1* and translocate calreticulin, and promote T cell dependent tumor inhibition. Moreover, the enhancement of dendritic cells and the activation and infiltration of T cells promote the improvement of tumor immune microenvironment. Another recent study reported that the new chemotherapeutic compound *TPH104* can induce ICD in BC cells by increasing the stimulating ability of dendritic cells, thereby enhancing tumor immunogenicity (Tukaramrao et al., 2021). The reports of these studies elaborated the role and potential target value of ICD in the process of BC.

Additionally, the study of ICD is not limited to BC, but also includes gastric cancer (Xiao et al., 2022), colorectal cancer (Wang et al., 2022), melanoma (Ren et al., 2022), neuroblastoma (Wang-Bishop et al., 2020), esophageal squamous cell carcinoma (Luo et al., 2022), etc. The latest research, a melanoma prognosis risk model consisting of three ICD genes was constructed to stratify the prognosis, immune cell infiltration, and immune related pathways of melanoma patients, and to screen the precise immunotherapy scheme (Ren et al., 2022). This is consistent with our research ideas. To explore the biomarkers of ICD in the tumor immune microenvironment, which can be used to regulate tumor immunosuppression to enhance patients’ anti-tumor immunity and promote the development of cancer immunotherapy.

ICD triggered by cancer therapy remodels the tumor immune microenvironment, mainly through the display or release of damage-associated molecular patterns by stressed and dead tumor cells, thereby enabling T-cell activation and the initiation of immune responses (Workenhe et al., 2021). Damage-associated molecular patterns can stimulate pattern recognition receptors of dendritic cells and T cells and promote the generation of primary immunity (Ahmed and Tait, 2020). It is worth mentioning that we comprehensively analyzed the correlation between the immune microenvironment of patients with BC and the ICD prognosis risk model using ESTIMATE, CIBERSORT, and TIMER. The results showed that the ICD risk model score was significantly negatively correlated with B cells, CD4 T cells, CD8 T cells, and dendritic cells. It has been confirmed that these cells are highly enriched in cancer, which can predict an improvement in the clinical prognosis of cancer (Bruni et al., 2020; Baxevanis et al., 2021). This is also consistent with our ICD risk model, in which high-risk groups of patients with BC are predicted to have poor survival and prognosis.

In summary, this study focused on the correlation between ICD subtypes and the tumor immune microenvironment in BC. These findings may be helpful in identifying anti-tumor immune regulation and immunotherapy targets in patients with BC for tumor control. In addition, we constructed and validated a risk model of ICD-related genes, which will provide an important theoretical basis for the survival outcome of clinical patients with BC and promote the development of precision immunotherapy for BC.

This study has some limitations that should be acknowledged. Firstly, due to the clinical data of the validation cohort lacks information about the progression of BC patients, such as tumor stage, we did not further analyze the correlation between the ICD risk model and clinical outcomes in the validation set. Besides, the BC sample data used in this study was downloaded from open database. More prospective studies are needed to be conducted to further confirm the prognostic value of ICD genes in BC.

## Conclusion

In conclusion, after comprehensive analysis and screening of ICD-related genes in BC patients, a prognostic risk model was constructed based on seven ICD-related genes (*ATG5*, *HSP90AA1*, *PIK3CA*, *EIF2AK3*, *MYD88*, *IL1R1*, and *CD8A*). The potential relationship between the risk model and the clinical characteristics, tumor immune microenvironment and survival status of BC was explored. This study provides new insights into the role of ICD in BC. The novel classification risk model based on ICD in BC established in this study can aid in estimating the potential prognosis of patients with BC and the clinical outcomes of immunotherapy and postulates targets that are more useful in comprehensive treatment strategies.

## Data Availability

The original contributions presented in the study are included in the article/Supplementary Material, further inquiries can be directed to the corresponding author.
